# Developing healthy attitudes to evidence through the Health Determinants Research Collaborations (HDRCs)

**DOI:** 10.1016/j.puhip.2025.100627

**Published:** 2025-06-16

**Authors:** Simon E. Kolstoe

**Affiliations:** University of Portsmouth, Portsmouth, PO1 2UP, UK

Dear Editor,

I enjoyed reading the paper “*Local authorities need tailored research ethics processes to* support *research capacity building*” by Levitas et al. [1] as it highlighted the importance of research ethics as a “*pre-requisite of high-quality research*”, and the relative immaturity of understanding relating to research ethics in the local authority setting.

The topic is timely, certainly in the UK, due to the significant funding that has been given to thirty local authorities through the National Institute for Health Research's (NIHR) grant programme: “*Health Determinants Research Collaborations (HDRC)*”, where the aim of the programme is to: *“… boost research capacity and capability within local government …*” so as to “*… create an evidence-informed culture within local government”* [2].

Clearly research has been identified by the NIHR as a way of creating good evidence, and informing good decision making. However, this then raises two questions: “*What makes good research?*”, and secondly: “*How is research evidence being used, by whom, and for which decisions?*”

In a 1994 editorial in the British Medical Journal the late Doug Altman, one of the most cited academics of all time, argued: “*We need less research, better research, and research done for the right reasons*” [3]. A few years later Sir Ian Chalmers and Prof Paul Glasziou, this time writing in the Lancet, claimed that “*85 % of research is wasted, usually because it asks the wrong questions, is badly designed, not published or poorly reported.*” They spent the next few years highlighting aspects of the research process that needed improving, or at least better understanding by the research community, so as to ensure higher quality *“Evidence Based Research”*. Although they did originally highlight research governance processes (such as ethics committees) as a source of research waste, a key conclusion coming from their work was that better research governance and ethics processes can be a solution to the problem of research waste and thus ultimately “*improve human health*” [4].

As a consequence of this line of thinking, if the NIHR's HDRC programmes are to create research capacity that generates good research for good decision making, I strongly agree with Levitas et al. that a mature research ethics process is vital. However, I disagree with the implication in their paper that the context of research in the local authority setting is so different from research conducted in universities and healthcare/medical settings that new or different ethics processes are needed. Indeed, many of the comments from their participants, and thus overall findings of their work, reflect widespread misunderstandings as to the role and function of research ethics, alongside queries as to the inclusion criteria for the type of work that might benefit from an ethics review. Far from being unique, these questions are identical to those frequently asked by the wider research community [5,6].

In agreement with Levitas et al., I do accept that one encouragement within the HDRC programmes is to develop ways of including local communities in designing and conducting research, while also including and enabling employees of local authorities with little prior research experience. Although this initiative is clearly important – as it is only really local communities and people dealing with them directly who have an authentic understanding of the health problems that affect them - research is difficult to get right even for experienced researchers, making it vital that research teams in local authorities receive appropriate support through effective ethics processes.

The solution is clearly for local authorities to work with universities through their HDRC programmes to establish these effective research ethics groups. But rather than reinventing the wheel, they need to build on the experience already present in universities and also nationally such as through the wealth of guidance and experience available from the Health Research Authority (among others). While it is certainly an accurate observation that research ethics and governance processes were originally developed within medical research due to the capacity of research in this area to cause significant harm, this does not mean that the experience of healthcare/medical research is irrelevant to research conducted by local authorities. There are undoubtedly different models in different settings, but over recent years medical research has become increasingly interdisciplinary, qualitative, and involving members of the public and patients. While researchers (and grant holders) are often incentivised to highlight the “novelty” of their work, this should not be at the expense of learning from others who may already have very workable solutions. We know what makes an effective ethics process, so now it is about applying what we know works into the local authority setting.
